# N^6^-methyladenosine (m^6^A) in pancreatic cancer: Regulatory mechanisms and future direction

**DOI:** 10.7150/ijbs.60115

**Published:** 2021-06-04

**Authors:** Jian Li, Fangjuan Wang, Yongkang Liu, Huaizhi Wang, Bing Ni

**Affiliations:** 1 Department of Pathophysiology, College of High Altitude, Army Medical University (Third Military Medical University), Chongqing 400038, PR China.; 2 Department of General Surgery, Air Force Hospital of Western Theater Command, Chengdu 610021, PR China.; 3 Department of Cardiology, Southwest Hospital, Army Medical University (Third Military Medical University), Chongqing 400038, PR China.; 4 Institute of Hepatopancreatobiliary Surgery, Chongqing General Hospital, University of Chinese Academy of Sciences, Chongqing 401120, PR China.

**Keywords:** m^6^A, pancreatic cancer, RNA modification, clinical application

## Abstract

N^6^-methyladenosine (m^6^A), the most abundant RNA modification in eukaryotes, plays a pivotal role in regulating many cellular and biological processes. Aberrant m^6^A modification has recently been involved in carcinogenesis in various cancers, including pancreatic cancer. Pancreatic cancer is one of the deadliest cancers. It is a heterogeneous malignant disease characterized by a plethora of diverse genetic and epigenetic events. Increasing evidence suggests that dysregulation of m^6^A regulatory factors, such as methyltransferases, demethylases, and m^6^A-binding proteins, profoundly affects the development and progression of pancreatic cancer. In addition, m^6^A regulators and m^6^A target transcripts may be promising early diagnostic and prognostic cancer biomarkers, as well as therapeutic targets. In this review, we highlight the biological functions and mechanisms of m^6^A in pancreatic cancer and discuss the potential of m^6^A modification in clinical applications.

## Introduction

Pancreatic cancer is one of the deadliest malignances with high invasiveness, early metastasis, lack of specific symptoms, and a 5-year survival rate of around 10% in the USA 1. In the past 20 years, the morbidity of pancreatic cancer has increased 6-fold in China 2. Pancreatic cancer risk factors include family history, smoking, type 2 diabetes, and obesity. Pancreatic ductal adenocarcinoma (PDAC) is the primary pathological type of pancreatic cancer. More than 85% of PDAC cases are complicated by distant metastasis, and patients present with poor clinical outcomes 3. Research efforts in PDAC have traditionally focused on genetic abnormalities, including chromosome gain/loss and somatic mutations. Previous research on these genetic alterations showed common mechanisms of pancreatic cancer tumorigenesis, such as activation of *KRAS* mutations or inactivation of tumor suppressor genes *TP53*, *SMAD4*, and *CDKN2A* 4. *KRAS*, which functions as a signal transducer between cell membrane-based growth factor signaling and the MAPK pathways, is the most frequently mutated oncogene (~ 90% of pancreatic cancer ) 5, 6. Somatic mutations in *TP53* tumor suppressor genes are also frequently observed in pancreatic cancer. The protein encoded by *TP53* plays a crucial role in multicellular organisms, where it prevents tumor formation 5, 6. Currently, surgical resection is the only treatment for pancreatic cancer, and adjuvant chemotherapy after surgical resection is an essential part of multimodality pancreatic cancer treatment. Unfortunately, although the prognosis of advanced pancreatic cancer has been enhanced by 5-fluorouracil/leucovorin with irinotecan and oxaliplatin (FOLFIRINOX) and gemcitabine/nab-paclitaxel, the development of chemoresistance in patients still results in poor clinical outcomes 7. It is worth noting that in addition to the restrictive contribution of genetic variation, the acquisition of chemoresistant phenotypes is usually largely reversible. The dynamic characteristics of cell plasticity and chemoresistance indicate that epigenetic alterations may be involved in the regulation of pancreatic cancer phenotypic heterogeneity 8. Importantly, in contrast to genetic defects, epigenetic alterations are reversible; therefore, they can be used as potential bona fide targets.

Accumulating evidence has revealed that epigenetic deregulation is critically associated with the pathophysiology of pancreatic cancer 9. N^6^-methyladenosine (m^6^A), methylated adenosine at the N^6^ position, is a new frontier of this field. Since its discovery in the 1970s 10, m^6^A has been identified as the most abundant form of internal mRNA modification in eukaryotes. Transcriptome-wide research has shown that m^6^A may affect more than 7000 transcripts in humans 11. m^6^A modifications can regulate the generation and function of transfer RNA (tRNA), ribosomal RNA (rRNA), and various non-coding RNAs (ncRNAs), such as microRNAs (miRNAs), long non-coding RNAs (lncRNAs), and circular RNAs (circRNAs) 12-14. Owing to improvements in molecular biology and sequencing, m^6^A modification has gained renewed interest in the past couple of decades and is currently the most widely studied type of RNA modification. Accumulating evidence has revealed that m^6^A affects almost every step of RNA metabolism, including alternative splicing, nuclear export, stability, translation, and decay 12, 15, 16. m^6^A are clustered in the 3′ untranslated region (UTR) near the stop codons of mRNAs and the m6A position has a consensus sequence of RRm6ACH (where R = G or A, and H = A, C or U) 11, 17. Recent studies have found that m^6^A modification is involved in various physiological behaviors, and its dysregulation may be implicated in the mechanisms associated with a variety of diseases 18-20. Increasing evidence suggests that m^6^A modification pathways are also implicated in the carcinogenesis of various malignancies, including pancreatic cancer 21-25. Aberrant regulation of m^6^A modification in coding and non-coding RNAs found in pancreatic cancer is crucial for multiple biological processes such as tumorigenesis, chemoresistance, and progression 24, 25. Here, we provide a comprehensive review of m^6^A modifications and highlight the potential molecular mechanisms of m^6^A in pancreatic cancer. We further emphasize the prospects for using m6A modification as a new biomarker and therapeutic target for pancreatic cancer.

## m^6^A writers, erasers, and readers

The installation of m^6^A modification is a reversible process modulated by the dynamic balance of m^6^A writer and eraser enzymes. The m^6^A writer complex, traditionally identified as a highly conserved multicomponent m^6^A methyltransferase complex (MTC), consists of methyltransferase-like 3 and 14 proteins (METTL3 and METTL14) and their cofactors Wilms' tumor 1-associating protein (WTAP). Several co-factors identified to interact with the MTC to affect m^6^A deposition include vir-like m^6^A methyltransferase-associated (KIAA1429), RNA-binding motif protein 15/15B (RBM15/15B), zinc finger CCCH domain-containing protein 13 (ZC3H13), and Fl(2)d-associated complex component (Flacc) 26-28. In addition, certain m^6^A methyltransferases do not exert their function via the MTC, including methyltransferase-like 16 (METTL16), zinc finger CCHC-type containing 4 (ZCCHC4), and methyltransferase-like 5 (METTL5) 29-31. METTL16 has recently been identified as an independent RNA methyltransferase and is responsible for m^6^A of mRNA in the 3′ UTR and A43 of the U6 small nuclear RNA (snRNA) during splicing 29, 32, 33. The demethylation of m^6^A is mediated by m^6^A erasers, mainly including fat mass and obesity-associated protein (FTO) and alkB homolog 5 (ALKBH5) 34, 35. FTO was identified as the first demethylase in nuclear RNA in 2011 34, and the notion of reversible m^6^A modification was described. Both FTO and ALKBH5 belong to the same protein family. However, studies have shown that FTO can act as a demethylase for both internal m^6^A and 5′ cap N^6^, 2Odimethyladenosine (m^6^Am) in mRNA 36, 37. Unlike FTO, ALKBH5 seems to be an m^6^A-specific demethylase that catalyzes the direct removal of m^6^A modification 35. In addition, ALKBH3 was recently identified as a novel demethylase of m^6^A in tumor progression via RNA demethylation and enhanced protein synthesis 38. ALKBH3 has also been shown to be an antitumor target and can be a potential diagnostic marker for cancer 39. The dynamic balance between m^6^A methylation and demethylation is essential for normal biological processes.

**Figure 1:**
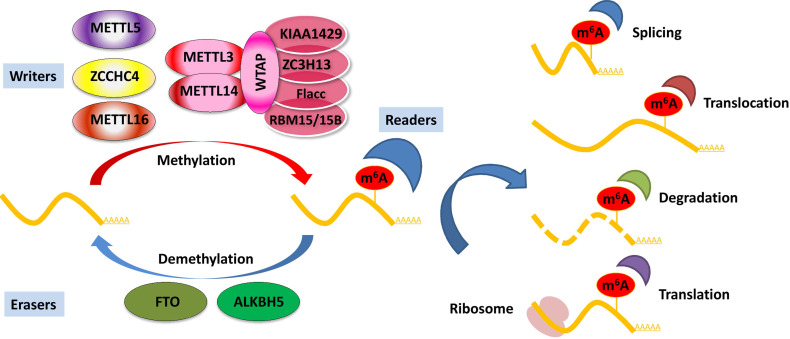
**Regulation of m^6^A modification on mRNA.** The m^6^A modification is added by writers, multicomponent methyltransferase complex (including METTL3, METTL14, WTAP, KIAA1429, ZC3H13, Flacc, and RBM15/15B) or METTL16, ZCCHC4, and METTL5 alone. m^6^A could be reversibly removed by m^6^A eraser proteins (FTO and ALKBH5) or recognized by m^6^A binding proteins (readers) to influence RNA splicing, translocation, stabilization, and translation.

Deposited on native RNA transcripts, m^6^A modification requires m^6^A-binding proteins (readers) to perform specific cellular bioprocesses. There are currently three main types of reader proteins, including the YT521-B homology (YTH) domain family proteins, heterogeneous nuclear ribonucleoproteins, and IGFBP family proteins. YTH domain-containing proteins, including YTHDF1, YTHDF2, YTHDF3, YTHDC1, and YTHDC2, were the first five characterized readers possessing a conserved domain for m^6^A recognition 40. YTHDF2 was the first identified among these and is the most studied m^6^A-binding protein. Nuclear YTHDF2 preserves the m^6^A modification of mRNA located in the 5′ UTR and influences mRNA translation under heat shock stress 41. In addition, YTHDF2 destabilizes m^6^A-containing RNA by recruiting the CCR4-NOT deadenylase complex in mammalian cells 42. Another m^6^A reader protein, YTHDF1, positively interacts with translation machinery and increases translation efficiency, eventually promoting protein synthesis 43. Furthermore, YTHDF3 can enhance protein synthesis in synergy with YTHDF1, and regulate m^6^A-modified mRNA decay mediated through YTHDF2 44. These three YTHDF proteins play crucial roles in modulating the translation and decay of m^6^A-modified mRNA in the cytoplasm 44. Heterogeneous nuclear ribonucleoproteins (hnRNPC, hnRNPG, and hnRNPA2B1) and IGFBP family proteins (IGFBP1-3) can act as m^6^A switch readers via remodeling specific m^6^A-dependent RNA structures and affect RNA-protein interactions for biological regulation 45-47. Notably, a number of novel m^6^A reader proteins have been identified in recent studies. Eukaryotic initiation factor 3 (eIF3) directly binds to the m^6^A site in the 5′ UTR of RNA, which is sufficient to recruit the 43S complex to initiate cap-independent translation 48. Fragile X mental retardation protein (FMRP) has been recently identified as an indirect reader and can regulate the stability of its m^6^A-modified mRNA targets via YTHDF2 49. Indeed, the aforementioned m^6^A reader proteins have pleiotropic functions and are implicated in regulating RNA splicing, translocation, stability, and translation, which affect various bioprocesses and are crucial in mammals (**Figure 1**).

## Aberrant m^6^A regulation in cancers

Increasing evidence has demonstrated that m^6^A modification is closely associated with tumor initiation and progression 12. Alterations in m^6^A levels are critical for cancer stem cell formation, cancer initiation, cancer metabolism, epithelial-mesenchymal transition (EMT), drug resistance, and cancer relapse 12, 50, 51. Studies have reported that METTL3 overexpression suppresses the self-renewal and oncogenesis of glioblastoma stem cells (GSCs) by increasing m^6^A levels and decreasing the expression of ADAM19, which plays crucial roles in GSCs 52. Another study showed that downregulation of METTL3 decreased m^6^A levels and restrained cancer migration, invasion, and EMT both *in vitro* and* in vivo*, and further confirmed that Snail, a key transcription factor of EMT, participates in m^6^A-mediated EMT 53. Notably, the global m^6^A abundance and expression of m^6^A modulators are highly heterogeneous, which indicates that the effects of m^6^A may vary in different cancer environments. Recently, it has been reported that the global m^6^A profile in a number of tumors abnormally decreases or increases, which may be related to the development and clinical outcome of cancer. For example, m^6^A levels were higher in approximately 70% of pancreatic cancer tissues than in pair-matched adjacent tissues, and higher levels of m^6^A were significantly correlated with decreased survival 24. Another group found that the level of m^6^A RNA was significantly elevated in human gastric cancer tissues compared with normal control tissues 54. Conversely, it has been reported that global m^6^A modification was substantially reduced in bladder cancer tissues, especially in advanced-stage bladder cancer patients. In addition, lower m^6^A modification content was related to poor clinical outcomes in patients with bladder cancer 55.

In addition, different components of m^6^A regulators have been shown to play either oncogenic or tumor-suppressive roles during tumorigenesis. For example, most studies support the oncogenic role of METTL3 in human cancers 13; however, METTL3 exerts a tumor-suppressive role in endometrial cancer 56. Although METTL14, another writer protein, was identified mainly as a tumor suppressor in cancers 13, it functions as an oncogene in acute myeloid leukemia 57 and breast cancer 58. The pleiotropic roles of METTL14 and other m^6^A writers are often inconsistent. Furthermore, m^6^A demethylase ALKBH5 plays a tumor-promoting role in the majority of studies 13; in contrast, ALKBH5 acts as a tumor suppressor in bladder cancer 59 and pancreatic cancer 22, 23. Even the same m^6^A regulator can play a controversial role in the same tumor. For instance, a different role of METTL3 in glioblastoma (GBM) has been shown in different studies 52, 60, 61. This may be due to the different sources of original samples used, different m^6^A sites, and m^6^A modified RNAs in different groups, resulting in tumor heterogeneity. Further research is required to clarify the enigmatic role of m^6^A modifications and m^6^A modulators in different cancer types and ultimately reconcile these seemingly contradictory findings in the future.

## Effects and underlying mechanisms of m^6^A in pancreatic cancer

**Figure 2:**
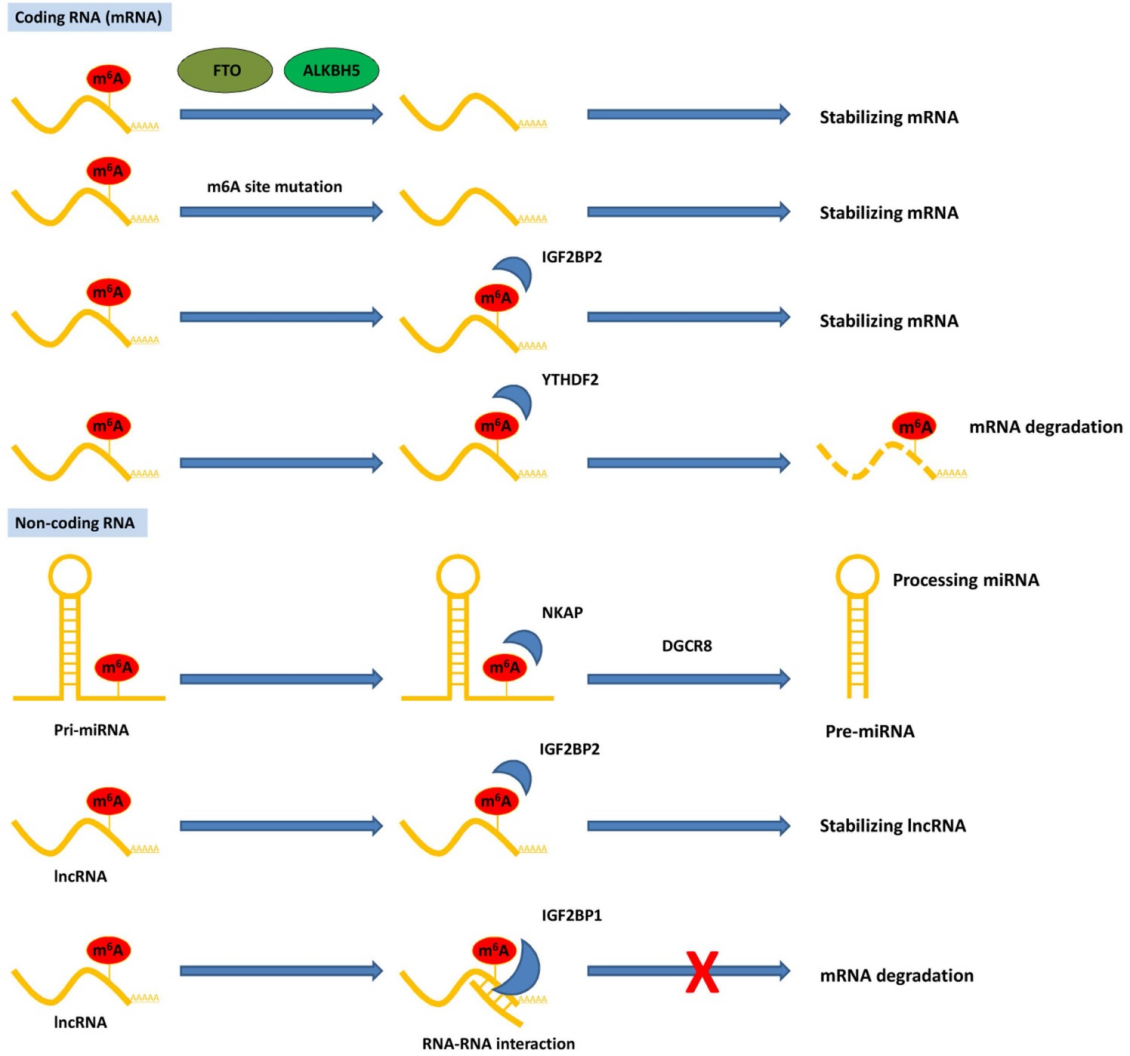
** Functions of m^6^A modification on coding and non-coding RNAs in pancreatic cancer.** The m^6^A modification recognized by reader proteins affects RNAs fate and exerts post-transcriptional regulation.

Although studies on the role of m^6^A in pancreatic cancer are in their early stages, emerging data have suggested that RNA m^6^A methylation is closely involved in pancreatic cancer progression, including carcinogenesis, proliferation, migration, invasion, EMT, and therapy resistance. Aberrant regulation of m^6^A and its modulators, including writers, erasers, and readers, plays a substantial role in pancreatic cancer by targeting various RNAs and signaling pathways (**Table 1**). The potential mechanism of m^6^A modification of coding and non-coding RNAs in pancreatic cancer is summarized in **Figure 2**. Herein, we briefly review recent studies on m^6^A modifications in pancreatic cancer.

### Dysregulation of m^6^A writers in pancreatic cancer

Aberrant expression of m^6^A writers in pancreatic cancer has been shown to play a crucial role in chemoresistance and progression. It has been reported that the m^6^A writer METTL3 is significantly overexpressed in pancreatic cancer and is linked to cancer aggressiveness and patient survival. Furthermore, METTL3 knockdown decreases m^6^A modifications and inhibits pancreatic cancer cell proliferation and migration 62. Taketo et al. showed that downregulation of METTL3 increased pancreatic cancer cell sensitivity to anticancer reagents, such as 5-fluorouracil, gemcitabine, cisplatin, and irradiation. Using cDNA expression analysis, METTL3 was involved in MAPK cascades, ubiquitin-dependent processes, RNA splicing, and cellular processes regulation 21. Furthermore, Zhang et al. reported that oncogenic miR-25 was excessively maturated by cigarette smoke condensate (CSC) through m^6^A modification, which is mediated by the upregulation of METTL3 expression in pancreatic duct epithelial cells. Interestingly, they found a coincidence of CSC-induced upregulation of miR-25 and METTL3, but not METTL14 and WTAP 25. However, the prognostic value was based on a small patient specimen size, and large-scale patient cohorts from multiple centers are needed to confirm the prognostic role of METTL3 in pancreatic cancer.

METTL14, another vital component of the m^6^A writer complex, has been identified as a tumor suppressor in multiple types of malignancies. In contrast, Wang et al. reported that upregulation of METTL14 directly targets the downstream PERP mRNA (p53 effector related to PMP-22) in an m^6^A-dependent manner, promoting the growth and metastasis of pancreatic cancer. Functionally, methylation of the target adenosine results in enhanced PERP mRNA turnover, thereby hindering PERP mRNA and protein expression 24. Another study showed that high METTL14 expression in pancreatic cancer tissues is associated with clinicopathological variables. Loss of METTL14 increased apoptosis induced by cisplatin in pancreatic cancer cells, and autophagy was enhanced through an mTOR signaling-dependent pathway 63. WTAP, a specific WT1-binding protein, has gained increasing attention owing to its important roles in tumorigenesis 64-66. In pancreatic cancer, Li et al. showed that high nuclear expression of WTAP was significantly correlated with poor prognosis, as well as several pathological characteristics 67. Further studies have shown that WTAP promotes metastasis and chemoresistance to gemcitabine via stabilizing Fak mRNA, and a specific FAK inhibitor, GSK2256098, could restore WTAP-mediated chemoresistance and metastasis in pancreatic cancer 68. However, as a regulatory factor of the m^6^A methyltransferase complex, there are still limited studies investigating the m^6^A modification-related function of WTAP in pancreatic cancer, which has to be clarified in the future.

At present, the research of m6A writers has mainly focused on METTL3 or METLL14; however, few studies have been mediated by METTL16. Our group recently revealed that METTL16 was significantly downregulated in pancreatic cancer and low METTL16 expression was a poor prognostic factor. In addition, METTL16 inhibited the p21 pathway by mediating m^6^A modification, resulting in a tumor-suppressive role in the proliferation of pancreatic cancer cells. Therefore, METTL16 may be a new therapeutic target for pancreatic cancer 69. However, more investigations are urgently warranted to fully characterize the function of METTL16 in pancreatic and other cancers.

### Dysregulation of m^6^A erasers in pancreatic cancer

Aberrant regulation of m^6^A erasers has also been demonstrated in pancreatic cancer. In a retrospective multicohort study, Cho et al. reported that ALKBH5 expression was positively associated with the prognosis of pancreatic cancer, and multivariate analysis showed that ALKBH5 is an independent prognostic factor 70. A recent study found that ALKBH5 was decreased in pancreatic cancer cells and inhibited pancreatic cancer motility by demethylating the lncRNA KCNK15-AS1 71. In addition, Guo et al. reported that knockdown of ALKBH5 increased pancreatic cancer cell proliferation, migration, and invasion *in vitro* and* in vivo*, whereas ALKBH5 overexpression restrained pancreatic cancer progression. Mechanistically, ALKBH5 inhibited PER1-ATM-CHK2-P53/CDC25C signaling in an m^6^A-YTHDF2-dependent manner, and P53-induced ALKBH5 activation acted as a feedback loop modulating m^6^A modification in pancreatic cancer 22. Gemcitabine resistance usually develops within weeks after starting treatment, limiting its overall efficacy as a first-line chemotherapy for pancreatic cancer. Another group showed that ALKBH5 is downregulated in the gemcitabine-treated patient-derived xenograft (PDX) model, and its upregulation enhanced PDAC cells to chemotherapy. Furthermore, ALKBH5 impaired the Wnt pathway and its downstream targets via demethylation of m^6^A-modified Wnt inhibitory factor 1 (WIF-1) transcripts 23. Based on the above knowledge, we could reach a firm conclusion that ALKBH5 acts as a tumor suppressor in pancreatic cancer.

Another m^6^A demethylase, FTO, previously linked with obesity and type II diabetes, was gradually discovered to be involved in diverse cancers. Tang et al. reported that FTO was overexpressed in pancreatic cancer, and knockdown of FTO decreased proliferation and promoted apoptosis of pancreatic cancer cells. Functionally, FTO has been shown to interact with the MYC proto-oncogene and bHLH transcription factor and regulate its stability via decreased m^6^A modification 72. Previous evidence has shown that FTO is strongly involved in the pathophysiology of various types of malignancies 73-75. The role of FTO in pancreatic cancer is not well understood and needs to be clarified in the future.

### Dysregulation of m^6^A readers in pancreatic cancer

Most of the biological functions of m^6^A are mediated by multiple m^6^A readers, which are also involved in pancreatic cancer. Chen et al. showed that YTHDF2 was overexpressed in pancreatic cancer, which was correlated with the later stages of pancreatic cancer. Furthermore, YTHDF2 orchestrated two cellular processes, including promoting proliferation and suppressing migration and invasion in pancreatic cancer cells, a phenomenon called the “migration-proliferation dichotomy”. Mechanistically, loss of YTHDF2 noticeably increased total YAP expression but suppressed TGF-β/Smad signaling 76. Another study showed that demethylase ALKBH5 suppressed pancreatic cancer progression by post-transcriptional activation of PER1 in an m^6^A-YTHDF2-dependent manner. PER1 mRNA was a novel target of YTHDF2, and downregulation of YTHDF2 enhanced PER1 mRNA expression 22. In addition, a recent study revealed that the rs142933486 G>T polymorphism in PIK3CB is significantly correlated with the clinical severity of PDAC patients by decreasing the PIK3CB m^6^A modification and causing a YTHDF2-mediated increase in its mRNA and protein levels. Interestingly, YTHDF2 predominantly binds to PIK3CB [G] compared to PIK3CB [T] in pancreatic cancer cells 77.

In addition to the YTH domain family proteins, IGF2BP proteins have also been identified as m^6^A readers. The primary role of IGF2BP2 is to modulate cell metabolism; however, emerging studies have shown that IGF2BP2 is involved in various types of cancers 78-80. Dahlem et al. reported that IGF2BP2 was markedly overexpressed in pancreatic intraepithelial neoplasia (PanIN), a well-known precursor of PDAC, implying that IGF2BP2 might be a diagnostic marker for early-stage pancreatic cancer. In addition, increased IGF2BP2 expression was associated with a poor prognosis in pancreatic cancer. Strict correlation analysis showed 22 highly positive genes and 9 genes negatively associated with IGF2BP2, and these genes were thought to be involved in apoptosis, ubiquitination, and the protein kinase C (PKC) signaling pathway. Interestingly, higher IGF2BP2 expression was detected in circulating tumor cells than normal hematological cells and normal tissues from the tumor origin 81. Similarly, another study showed that IGF2BP2 was correlated with clinical outcome and multiple biological processes involved in cancer, of which the most significant processes were associated with cancer cell cycle, immortalization, and tumor immunity 82. Another study also found that IGF2BP2 promoted cell proliferation and aerobic glycolysis in PDAC by directly binding and stabilizing GLUT1 mRNA 83. In addition, Schaeffer et al. reported that IGF2BP3 overexpression was associated with poor survival in PDAC 84. Another group demonstrated that IGF2BP3 and IGF2BP3-bound transcripts were localized in cytoplasmic RNA granules, and IGF2BP3 promoted pancreatic cancer cell migration and invasion by regulating the localized translation of IGF2BP3 target transcripts in cell protrusions 85. Furthermore, they found that loss of KIF20A suppressed the accumulation of IGF2BP3-containing stress granules in cell protrusions and restrained local protein expression from certain IGF2BP3-bound transcripts, ARF6 and ARHGEF4 86.

Emerging evidence has shown that m^6^A readers can regulate ncRNAs 13. Recently, NF-κB associated protein (NKAP) was identified as a reader of m^6^A in pri-miR-25 maturation, and mature miR-25 could promote pancreatic cancer progression 25. LncRNA differentiation antagonizes non-protein coding RNA (DANCR) involved in the tumorigenesis of different cancer types 87. IGF2BP2 acts as a reader for the m^6^A modification of DANCR and promotes pancreatic cancer cell proliferation 88. In turn, ncRNAs also regulate m^6^A reader proteins. Wan et al. revealed that IGF2BP1 is overexpressed and correlated with poor survival in pancreatic cancer. Additionally, IGF2BP1 is a new target of miR-494, and re-expression of miR-494 can partially reverse the oncogenic role of IGF2BP1 89. Xu et al. showed that IGF2BP2 was identified as a direct target of miR-141, and the miR-141/IGF2BP2 axis promoted pancreatic cancer cell proliferation by activating the PI3K/Akt pathway* in vitro* and *in vivo* 90. Another study reported that lncRNA NEAT1 was overexpressed and correlated with poor prognosis in patients with pancreatic cancer. Further, NEAT1 could increase the combination of E74 like ETS transcription factor 3 (ELF3) mRNA and IGF2BP1, therefore enhanced the stability of ELF3 mRNA 91. A recent study showed that LINC00261 is a tumor suppressor with clinical significance in pancreatic cancer. Mechanistically, LINC00261 could decrease c-myc mRNA stability by sequestering IGF2BP1 92. Furthermore, the rs7495G allele in the hnRNPC gene promotes hnRNPC expression by disrupting a putative binding site for miR-183-3p in pancreatic cancer 93. Dysregulation of m^6^A readers aberrantly regulates the expression of various RNAs and their downstream pathways, which play a crucial role in pancreatic cancer.

## Mutants of m^6^A sites and m^6^A regulators in pancreatic cancer

Mutations in tumor-promoting and tumor-suppressing genes are common during tumor development. Nevertheless, little is known about the role of mutations at the m^6^A site in cancer. m^6^A site mutations may influence RNA m^6^A modification, leading to aberrant post-transcriptional regulation and participation in tumorigenesis. For example, a recent study showed that m^6^A at the point-mutated transited codon 273 (G>A) of p53 pre-mRNA enhanced its splicing via methylation of METTL3, resulting in overexpression of the p53 R273H mutant protein, which induced drug resistance in cancer cells 94. In pancreatic cancer, it was reported that the missense variant rs142933486 in the 20th exon of *PIK3CB* was clearly correlated with the clinical outcome. Further study identified that this variant was a G>T change and was coincidently located 3 bp from a predicted m^6^A site. *PIK3CB* [T] expression decreased PIK3CB m^6^A levels and enhanced its mRNA and protein expression. Functionally, upregulation of PIK3CB potentiates the proliferation and migration of PTEN-deficient pancreatic cancer cells by targeting the AKT signaling pathway 77.

Given the pivotal role of m^6^A modification in various biological processes, it is rational to assume that genetic variants in m^6^A regulators, including its writers, erasers, and readers, might be involved in oncogenesis. The m^6^A eraser FTO, identified by genome-wide association studies (GWAS), is a dangerous gene related to the risk of obesity and body mass index (BMI) 95, 96. In a case-control study, the FTO polymorphism rs9939609 was linked to the risk of pancreatic cancer in Japanese population 97. Furthermore, there was a significant association between pancreatic cancer and endometrial cancer; however, no statistical significance was found in other malignancies through a meta-analysis 98. Overall, rs9939609 in the *FTO* gene might be a potential biomarker for early diagnosis or gene therapy targeting pancreatic cancer. Interestingly, another study showed that *FTO* gene mutations might be positively correlated with pancreatic cancer only in overweight people. Stratification analysis revealed that both heterozygous and homozygous mutations of the *FTO* IVS1-27777 C>A and IVS1-23525 T>A SNPs were correlated with a decreased risk of pancreatic cancer among participants with BMIs <25 kg/m^2^ but were correlated with an increased risk among participants with BMIs >25 kg/m^2^ 99. In addition, to investigate all SNPs in 22 m^6^A modification genes, Ying et al. recently conducted a two-stage case-control study in a Chinese population and found that rs7495 in the 3′ UTR of hnRNPC, an m^6^A reader, was significantly linked to an increased risk of PDAC. Mechanistically, the rs7495G allele promoted hnRNPC expression by disrupting a putative binding site for miR-183-3p. 93.

## m^6^A as biomarkers of pancreatic cancer

Studies have shown that most m^6^A regulators are dysregulated in pancreatic cancer, and their expression was found to be correlated with clinical outcomes, suggesting the potential to become novel biomarkers for the early diagnosis and prognosis of pancreatic cancer. PanIN is a well-known precursor of PDAC, and early detection of PanIN would help block the progression of PanIN to PDAC. A recent study revealed that IGF2BP2 was significantly overexpressed in human PanIN 81, which is associated with a high risk of developing pancreatic cancer. Consistent with this result, Huang et al. reported that IGF2BP2 protein levels were gradually elevated from normal pancreas and PanIN to PDAC in mice 83. These findings highlight that IGF2BP2 has potential value for the early diagnosis of pancreatic cancer. In addition, the detection of circulating tumor cells (CTCs) is a blood-based, non-invasive approach that can be used for the early diagnosis of cancers 100, 101. A recent study showed that m^6^A levels in CTCs were significantly elevated in lung cancer patients, suggesting that the examination of m^6^A in CTCs might be a novel method for cancer diagnosis 102. However, the m^6^A modification of CTCs in pancreatic cancer is not well understood. Further studies should elucidate whether the dysregulation of m^6^A modification and m^6^A modulators is an early event in pancreatic cancer tumorigenesis, which is crucial for developing a potential approach for utilizing m^6^A and m^6^A regulatory factors for early cancer diagnosis.

Recently, several studies have investigated the prognostic value of m^6^A-related mRNA signature and m^6^A regulators in pancreatic cancer using database analysis 82, 103-106. For instance, Meng et al. provided an mRNA signature that may enhance the prognostic prediction of patients with pancreatic cancer based on the genetic status of m^6^A regulators. In addition, they generated a 16-mRNA signature score system via least absolute shrinkage and selection operator (LASSO) Cox regression analysis, and a high-risk score was clearly associated with poor prognosis 104. Similarly, 283 candidate m^6^A-related genes and 4 m^6^A regulators, including RBM15, METTL14, FTO, and ALKBH5, differed clearly among different stages of the American Joint Committee on Cancer (AJCC) staging system 103. Additionally, another study showed that m^6^A regulator-based sample clusters, including 19 m^6^A regulators, were associated with overall survival (OS), and LASSO regression identified a six-m^6^A-regulator-signature prognostic model, including METTL3, KIAA1429, HNRNPC, YTHDF1, IGF2BP2, and IGF2BP3 103. Furthermore, our group found that METTL16 was significantly decreased in pancreatic cancer and was correlated with patient survival, indicating the prognostic value of METTL16 in pancreatic cancer 69. As mentioned before, upregulation of ALKBH5 or IGF2BP2 were both significantly associated with poor survival in several studies, highlighting the prognostic value of ALKBH5 and IGF2BP2 in pancreatic cancer. Overall, the m^6^A regulatory factors and m^6^A-related mRNA signature, which correlate with clinical outcomes, can be implicated in the malignant progression of pancreatic cancer.

## m^6^A as a therapeutic target of pancreatic cancer

Based on the critical roles of m^6^A modification and m^6^A modulators in pancreatic cancer, m^6^A exhibits great potential as a novel therapeutic target. As mentioned above, downregulation of METTL3 suppressed proliferation, migration, and invasion and enhanced the sensitivity of pancreatic cancer cells to anticancer reagents 21. Downregulation of METTL14 suppresses pancreatic cancer cell growth and metastasis to the liver and increases apoptosis induced by cisplatin 24, 63. Furthermore, overexpression of ALKBH5 inhibits proliferation, migration, and invasion both* in vitro* and *in vivo* 22, 23. Thus, these results provide a strong rationale for m^6^A regulators to be potential therapeutic targets for pancreatic cancer therapy in the future. Recently, FTO has been the most attractive target for developing specific inhibitors targeting m^6^A modulators for cancer treatment. Meclofenamic acid (MA), a nonsteroidal anti-inflammatory drug, is a selective FTO inhibitor that competes with FTO binding sites and suppresses m^6^A demethylase activity 107. Another MA-derived inhibitor, FB23-2, that directly binds to FTO has been developed to impair proliferation and increase the differentiation of human acute myeloid leukemia cells *in vitro* and* in vivo* 108. Su et al. reported that R-2-hydroxyglutarate (R-2HG) inhibited FTO demethylase activity and increased m^6^A modification in leukemia cells, which decreased the stability of MYC/CEBPA transcripts, exhibiting broad anticancer activity* in vitro* and* in vivo* 109. Additionally, this group recently showed that R-2HG impaired aerobic glycolysis by targeting the FTO/m^6^A/PFKP/LDHB axis in leukemia 110. Of note, Peng et al. showed that Entacapone, an FDA-approved drug, could directly bind to FTO and inhibit FTO activity 111. FTO has been found to promote proliferation and decrease the apoptosis of pancreatic cancer cells 72, and the aforementioned FTO inhibitors might provide new therapeutic opportunities for pancreatic cancer patients.

Although immune checkpoint blockade (ICB) therapy is at the forefront of immunotherapy for various cancers, many patients do not respond or develop resistance to ICB 112, 113. In recent years, the critical role of m^6^A modification in regulating the immune response to anti-PD-1 therapy has been reported. Wang et al. found that inhibition of m^6^A modification by knockdown of METTL3 and METTL14 enhanced the immune response to anti-PD-1 treatment in mice 114. Another study showed that ALKBH5 enhanced anti-PD-1 therapy response by regulating Mct4/Slc16a3 expression and lactate content and the composition of tumor-infiltrating Tregs and myeloid-derived suppressor cells in the tumor microenvironment 115. In addition, Han et al. discovered that the knockdown of YTHDF1 increased the efficacy of PD-L1 ICB therapy 111. Interestingly, using a small-molecule ALKBH5 inhibitor could enhance the efficacy of cancer immunotherapy, suggesting future translational applications 115. Thus, the crucial role of m^6^A modification in regulating immune response may contribute to cancer immunotherapy, and further research is required to provide new directions for efficient pancreatic cancer treatment. Although targeting m^6^A appears to be a promising new therapeutic strategy, its side effects cannot be ignored. Since m^6^A plays a broad and critical role in almost all aspects of RNA metabolism, the application of specific agonists or inhibitors of m^6^A regulatory proteins may disturb normal physiological processes, resulting in severe outcomes.

## Conclusions and perspectives

RNA m^6^A modification has gained increasing attention as a new frontier of epigenetic research, and its involvement in a variety of biological processes and disease progression has been recently reported. This review summarizes recent advances in understanding the regulatory mechanisms and future direction of m^6^A in pancreatic cancer. The specific mechanism for m^6^A modification in pancreatic cancer, it should be noted, is complex and even contradictory among studies. For example, it seems inconsistent that the m^6^A writer MELLT3, which increases the m^6^A level, acts as an oncogene in pancreatic cancer, while the m^6^A eraser FTO, which reduces the m^6^A level, is also an oncogene in pancreatic cancer. It is hypothesized that if the writer and the eraser act on the same site of a particular RNA, they may conversely modify m^6^A and play opposite roles in cancer. This phenomenon has also been reported in a number of cancers, including colorectal cancer 116, 117 and breast cancer 118, 119. These seemingly contradictory findings may be attributed to various factors, such as intratumoral heterogeneity, different tumor origins, and ethnic groups. For instance, the rs9939609 polymorphism in FTO was significantly correlated with cancer risk in Asians, while no consistent association was found in Caucasians and mixed ethnicities 98. In addition, the m^6^A reader is a crucial effector of post-transcriptional regulation, which may explain the seemingly inconsistent role between m^6^A writers and m^6^A erasers. Furthermore, there are still potential m^6^A regulatory factors that have not been discovered thus far that may also be involved in m^6^A modification. Therefore, additional investigations are warranted to characterize the existence of other regulators implicated in m6A fully.

Accumulated studies have revealed the importance of m^6^A regulators as potential early diagnosis and prognosis biomarkers for pancreatic cancer. For example, overexpression of METTL3 has been associated with poor prognosis in pancreatic cancer 62. Several studies have reported that IGF2BP2 was markedly overexpressed in PanIN and associated with clinical outcomes, implying that IGF2BP2 might be a potential diagnostic and prognostic biomarker for pancreatic cancer 81-83. Intriguingly, we also found that both m^6^A writer (METTL3 and METTL14) and eraser (FTO) are abnormally upregulated and have a carcinogenic effect in pancreatic cancer. Therefore, to a certain extent, global m^6^A signatures may be unreliable for pancreatic cancer diagnosis, and the m^6^A modification of target genes or sites may be used as better biomarkers. However, it is reported that the main m^6^A detection methods currently available cannot accurately detect the m^6^A profile in the whole transcriptome, so it is difficult to fully understand the correlation between m^6^A modification and tumors 120. In addition, current detection methods still require a large amount of RNA and cannot accurately detect RNA modifications in rare and precious samples. Thus, a novel detection approach with reduced sample volume, high precision, and low cost is urgently needed. This will help promote the use of m^6^A target transcripts or m^6^A sites as potential novel biomarkers for pancreatic cancer. Of note, the biological function of m^6^A modification at specific sites still remains largely unclear. With the advancement of editing tools based on CRISPR, different m^6^A editing systems have been reported recently, which may greatly promote research on the effect of specific m^6^A modifications in the near future. For instance, the fusion of dCas9 or dCas13 with m^6^A writers or erasers can edit specific m^6^A sites guided by single-guide RNA (sgRNA) and the m^6^A protospacer adjacent motif (PAM) locus 121, 122. The m^6^A modification editing tool represents a revolutionary advancement in the study of m^6^A functions and seems to provide unprecedented opportunities for m^6^A research. Future research on m^6^A modification will focus on accurately identifying m^6^A sites using m^6^A editing tools to edit m^6^A and then conducting functional experiments at these specific m^6^A sites.

Apart from m^6^A, other RNA modifications have also been disclosed in pancreatic cancer, such as 5-methylcytosine (m^5^C), 3-methylcytosine (m^3^C), 1-methyladenosine (m^1^A), and 27-methylguanine (m^27^G). Yang et al. recently revealed that m^5^C methyltransferase NSUN6 is downregulated in pancreatic cancer and suppressed pancreatic cancer cell proliferation and tumor growth in xenograft mouse models. Notably, NSUN6 has also been reported to play an essential role in predicting pancreatic cancer recurrence and patient survival time 123. Another study showed that ALKBH3 is a 1-methyladenosine (m^1^A) and 3-methylcytidine (m^3^C) demethylase of transfer RNA (tRNA) and can increase cancer cell proliferation, migration, and invasion 124. Interestingly, a previous study reported that ALKBH3 was overexpressed in pancreatic cancer and was associated with advanced tumor status, pathological stage, and VEGF intensity. Thus, we have good reason to speculate that ALKBH3 may play an important role in pancreatic cancer by regulating RNA modification 125. In addition, it has been reported that m^1^A, m^27^G, and Asm are the most important features distinguishing cell lines derived from poorly differentiated pancreatic cancer from well-differentiated pancreatic cancer 126. Since the tumor differentiation state is related to the degree of cancer malignancy, m^1^A, m^27^G, and Asm may function as valuable biomarkers for pancreatic cancer. Notably, it has been reported that various RNA modifications, such as m^6^A and m^5^C, could regulate the same RNA and coordinately promote translation 127. Furthermore, Chen et al. recently identified potential cross-talk between m^6^A and m^5^C methylation at the multiomic level, which is also involved in onco-immunogenic features and patient survival across 33 cancer types 122. These results highlight multiple cross-talks of RNA modifications in cancers, which provide novel and essential insights into the epigenetic regulation of cancer and opens up new avenues for developing related therapeutic targets.

Although emerging evidence has shown that m^6^A modification plays essential and diverse biological roles in the development and progression of pancreatic cancer, m^6^A studies in pancreatic cancer are still incipient. We know very little about the detailed mechanism of m^6^A modification in pancreatic cancer, and the conclusions of some of the studies above are sometimes inconsistent in this field. Further research is needed to clarify the heterogeneity and complexity of m^6^A modifications and m^6^A modulators in the development of pancreatic cancer. More efforts are also needed in the future to identify specific m^6^A for the early diagnosis of cancer and to develop specific inhibitors to target m^6^A regulatory factors. The rapid development of m^6^A mapping methods and m^6^A editing tools will greatly promote the research of m^6^A at the single-nucleotide level, which may significantly promote the development of this exciting field. In general, research in this field is progressing rapidly. It is expected that research on m^6^A modification in pancreatic cancer will be greatly expanded in the near future.

## Figures and Tables

**Table 1:** Roles of m^6^A regulators in pancreatic cancer

m^6^A regulators	Expression	Roles in cancer	Biological function	Target/signaling	References
**Writer**					
METTL3	↑	oncogenic	promotes chemoresistance	MAPK cascades, ubiquitin-dependent process, and RNA splicing and regulation of cellular process	21
METTL3	↑	oncogenic	promotes proliferation, migration, and invasion	--	62
METTL3	↑	oncogenic	promotes initiation and progression	processing pri-miR-25	25
METTL14	↑	oncogenic	promotes growth and metastasis; increases apoptosis induced by cisplatin	PERP/increase mRNA turnover	24
METTL14	↑	oncogenic	inhibits apoptosis induced by cisplatin and autophagy	mTOR signaling pathway	63
WTAP	↑	oncogenic	promotes metastasis and chemoresistance to gemcitabine	stabilizing Fak mRNA	67
METTL16	↓	antitumor	inhibits proliferation	p21 pathway	69
**Eraser**					
ALKBH5	↓	antitumor	inhibits cell motility	KCNK15-AS1	71
ALKBH5	↓	antitumor	inhibits proliferation, migration, and invasion	PER1-ATM-CHK2-P53/CDC25C signaling	22
ALKBH5	↓	antitumor	inhibits proliferation, migration, and invasion	WIF-1/Wnt signaling	23
FTO	↑	oncogenic	promotes proliferation and inhibits apoptosis	MYC, bHLH/ regulate mRNA stability	72
**Reader**					
YTHDF2	↑	Oncogenic/ antitumor	promotes proliferation and suppresses migration, invasion, and EMT	YAP and TGF-β/Smad signaling	76
YTHDF2	↑	oncogenic	promotes proliferation, migration, and invasion	PER1/inhibit mRNA degradation	22
YTHDF2	↑	oncogenic	promotes proliferation and migration	PIK3CB	77
IGF2BP2	↑	oncogenic	involved in apoptosis and ubiquitination	PKC signaling pathway	81
IGF2BP2	↑	oncogenic	promotes proliferation and aerobic glycolysis	GLUT1	83
IGF2BP2	↑	oncogenic	promotes proliferation	PI3K/AKTpathway	90
IGF2BP2	↑	oncogenic	promotes proliferation	lncRNA DANCR	87
IGF2BP1	↑	oncogenic	promotes proliferation	AKT signaling pathway	89
IGF2BP1	↑	oncogenic	promotes proliferation and metastasis	ELF3	91
IGF2BP1	↑	oncogenic	promotes proliferation	c-myc	92
IGF2BP3	↑	oncogenic	promotes migration and invasion	ARF6 and ARHGEF4	86
hnRNPC	↑	oncogenic	promotes proliferation	miR-183-3p	93
